# Logged in, not lagging behind: A purpose-oriented use of technology for youth health

**DOI:** 10.1371/journal.pdig.0000881

**Published:** 2025-06-13

**Authors:** Matea Cañizares

**Affiliations:** Colegio Johannes Kepler, Quito, Ecuador; McGill University, CANADA

In our highly digitized planet, two in three people use the internet; however, the variety of digital skills people apply is limited, particularly in low- and middle-income countries (LMICs) [1]. In addition, young people feel disengaged from government decision-making when they distrust their politicians [2], thus becoming disempowered to act as advocates. As a result, where accountability is most needed, policy reforms may omit youth perspectives and, therefore, overlook issues that are important to this age group. Promoting digital citizenship for health (DC4H) could enable young people to contribute in meaningful ways to policy-making processes related to the health matters that affect them [3], even in resource-limited countries.

The concept of DC4H focuses on the impact of digital technology on health and the use of digital tools for information, advocacy, and participation, but could be more broadly understood to consider the ability of young people to produce knowledge on health issues using digital tools with the aim of informing policy. While access to the internet remains unequal between urban and rural areas, and between LMICs and high-income countries, even limited technology can be leveraged through free software and cloud-based platforms. I analyze here my experience bringing the results of my research on environmental determinants of health to the Ecuadorian government, as an example of how young people can develop DC4H in a middle-income country.

In Ecuador, young people do not feel that politicians listen to them [4]; there are policy instruments to promote the empowerment and participation of young people as actors in public policy, but these are not commonly implemented [5]. In addition, school students have expressed that they would like the curriculum to be modernized to become proficient in the use of digital tools [6]. Although solar radiation is an important health concern [7], the government does not have clear guidelines to protect young people from high solar radiation beyond alerts through the meteorological institute’s social media [8]. Reflecting on my lived experience playing football matches under the scorching sun, I looked for research on the potential negative consequences on human health, learned to use Google Earth Engine to assess the risks, and successfully approached the Ministry of Education with my results and recommendations [9].

**Fig 1:**
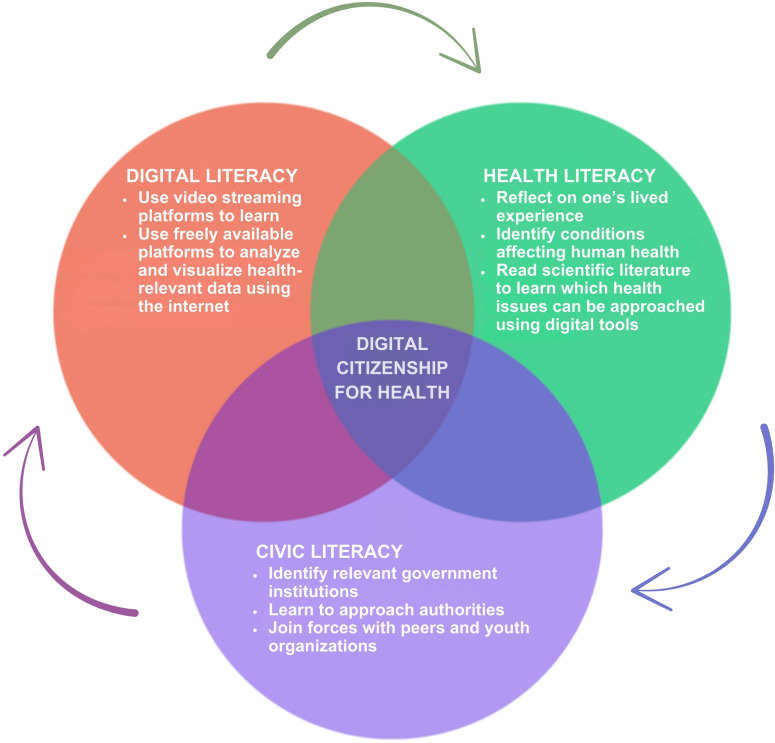
Practical application of the digital citizenship for health (DC4H) framework.

To develop digital, health, and civic literacies, exceptional resources and knowledge are not required (Fig 1). To develop health literacy, young people can begin by examining their lived experience, asking themselves what conditions affect their health on a daily basis, and which could be the biggest health issues among their peers, followed by reading literature online to identify which concerns can be approached using digital tools. To develop digital literacy, they can learn on a video streaming platform such as YouTube how to collect and explore health-relevant data using a cloud-based tool such as Google Earth Engine, which allows users to analyze and visualize environmental, climate, and water resources datasets [10]. To develop civic literacy, young people can learn to identify which government institutions are tasked with addressing specific determinants of health and how to approach them together with peers and with the support of youth organizations.

Young people have limited opportunities at school to learn about and apply digital tools; only 50% of lower secondary and 65% of upper secondary schools have an internet connection [1]. Still, having Wi-Fi does not guarantee the promotion of digital skills and learning [1]. Meanwhile, countries without computers in schools, such as Ecuador, have banned mobile phones from the premises, instead of leveraging their use for purpose-oriented activities. If only modest digital transformations hold the potential to improve educational outcomes [1], middle-income countries should reform the school curriculum to offer the space and flexibility to develop digital literacy. Through time and effort, by trial and error, young people can progressively learn to use their own devices to develop DC4H skills.

Through a purpose-oriented use of technology, an informed youth can progressively become more engaged and motivated to learn more about the determinants of health and collect and process information to participate in discussions and decision-making. Furthermore, their work can improve government transparency and accountability, thus creating the conditions for greater trust and, in consequence, more youth engagement.

Today’s youth are tomorrow’s decision-makers and policy reformers. If they are not meaningfully involved in the conversation today, they will learn that youth perspectives can be overlooked and replicate this practice as adults. To prevent this vicious cycle of youth being excluded from discussions and decisions on health matters that concern them the most, we need to support young people in taking fuller advantage of a digitized world. We can begin with the resources available to them and work towards enhancing their access to technology.
